# Improvement of Overall Skin Condition in Acne and Oily Prone Skin With an Amino Acid Cleanser Containing Salicylic Acid, Glucuronolactone, and Ceramides

**DOI:** 10.1111/jocd.70759

**Published:** 2026-02-26

**Authors:** Yunfei Ai Levin, Yansi Lyu, Xiaofeng He

**Affiliations:** ^1^ L'oréal Dermatological Beauty China CeraVe Shanghai China; ^2^ Department of Dermatology Shenzhen University General Hospital Shenzhen Guangdong China; ^3^ L'oréal China Research & Innovation Shanghai China

## Abstract

**Background:**

Cleansers and moisturizers are two of the most essential skincare categories, both of which have significant dermatological implications. However, most studies focus on the cleansing power of surfactants and their potential damage to the skin barrier. Few studies explore the combined effects of surfactants and active ingredients on skin condition.

**Objective:**

The main focus of this research was to explore the comprehensive improvement effects of a cleanser containing salicylic acid, glucuronolactone, and ceramides on acne and oily‐prone skin.

**Methods:**

A total of 42 volunteers used amino acid cleanser containing salicylic acid, glucuronolactone, and ceramides for 28 days. Noninvasive methods combined with dermatologist evaluation to detect skin barrier changes in TEWL, hydration, facial sebum content, acne counts, facial redness, and texture were used. Adverse reactions were also assessed.

**Results:**

The results indicate that after continuous use for 4 weeks, there was a significant improvement in skin barrier function, redness, moisture content, and skin texture, while acne‐related indicators also showed a significant reduction. No adverse events occurred during the entire testing process.

**Conclusion:**

In summary, an amino acid cleanser containing salicylic acid, glucuronolactone, and ceramides has anti‐acne, repairing, soothing, and texture‐improving properties, offering multidimensional improvement for acne and oily‐prone skin conditions.

## 1 Introduction

Cleansing is an essential daily practice that plays a significant role in both skin health and the management of skin conditions. Several multidimensional functions of skin cleansing were recognized and categorized into five distinct dimensions: hygienic and medical significance, sociocultural and interpersonal relevance, impact on mood, emotion, and well‐being, cosmetic and aesthetic benefits, and corneobiological interactions [1].

Previous studies have primarily focused on the immediate cleansing ability of cleansers to remove skin sebum and dirt [2, 3], as well as exploring the relationship between surfactants and skin proteins and lipids to minimize the impact on the skin barrier [4]. Recent studies have started to explore the broader effects of surfactants combined with active ingredients, with a primary focus on acne treatment, such as the effectiveness of salicylic acid‐containing cleansers for acne [5]. On the other hand, products on the China market, in addition to cleansing, have started to promote more beauty benefits, including repair and whitening. However, few studies explore the combined effects of surfactants and active ingredients on the overall skin condition, including anti‐acne, repair, and skin texture. Therefore, the main focus of this research was to explore the comprehensive improvement effects of a cleanser containing salicylic acid, glucuronolactone, and ceramides on acne and oily‐prone skin.

## 2 Materials and Methods

42 Chinese subjects were enrolled to take part in this randomized, 28‐day study. The inclusion criteria of subjects were as follows: Chinese males or females aged from 18 to 50 years; self‐reported as having combination or oily skin type, with 50% self‐reporting sensitive skin; facial acne Grades 2–3 (IGA scale), with ≥ 5 inflammatory acne lesions and ≥ 10 noninflammatory acne lesions; Sebumeter measurement in the forehead area (average of 3 measurements) > 100 μg/cm^2^. Exclusion criteria are as follows: having been diagnosed with known allergies to facial skin care products; breastfeeding, pregnant, or planning to become pregnant during the study according to subject self‐report. All subjects were informed and gave their consent before enrollment. The treatment cleanser is an amino acid surfactant‐based cleanser that contains 2% salicylic acid, 1.1% gluconolactone, 0.1% hectorite, niacinamide, and a blend of three essential ceramides, that is ceramide EOP, NP, and AP (ceramide 1,3,6‐II). Subjects used the product continuously for 28 days. The measurement time points were as follows: before product use (D0), immediately after product use (D0Timm), 2 h after product use (D0T2h), 4 h after product use (D0T4h), 8 h after product use (D0T8h), 7 days after product use (D7), 14 days after product use (D14), and 28 days after product use (D28). In addition, on Day 0 (baseline testing day), an appropriate amount (one pump, approximately 2–3 mL) of the test product was applied to the lower forehead area and one randomly assigned forehead area, then rinsed off. The contralateral forehead control area was cleansed with purified water only. Skin sebum level was then measured at both the test and control forehead areas immediately after cleansing, as well as at 4th hour and 8th hour post‐cleansing. Both the participants and investigators were blinded to the allocation of the treatment and control areas.

### 2.1 Instrument Assessment

The Sebumeter SM815 is used to measure the sebum content of the skin. A decrease in the measurement value indicates a reduction in the sebum content of the skin. The Tewameter TM Hex is used to measure the transepidermal water loss (TEWL) rate of the skin. A decrease in the measurement value indicates an improvement in the skin barrier. The Corneometer CM825 is used to measure the moisture content of the stratum corneum. An increase in the measurement value indicates an increase in the moisture content of the skin's stratum corneum.

### 2.2 Dermatologist Evaluation

Dermatologists evaluated the characteristics of various skin lesions (open comedone counts, closed comedone counts, total noninflammatory lesions, papule counts, pustule counts, total inflammatory lesions, skin lesion total counts) and the skin status (skin texture [tactile], skin texture [visual], skin tone evenness, redness, overall appearance of skin, IGA scale) of the subjects. Rating criteria are as follows: from 0 to 9 scale, where 0 indicates very good and 9 indicates very poor. IGA scale are as follows: from 0 to 4 rating system, where 0 indicates very good and 4 indicates very poor.

### 2.3 Statistical Analysis

SPSS 28.0 was used for data statistics. The test data are tested for normal distribution. If the test data are normally distributed, the *t*‐test method was used for statistical analysis. If the test data are non‐normally distributed, the rank sum test method was used for statistical analysis. The rank data were statistically analyzed by the rank sum test, which were statistically significant at *p* < 0.05.

## 3 Results

### 3.1 Instrument Assessment

**FIGURE 1:**
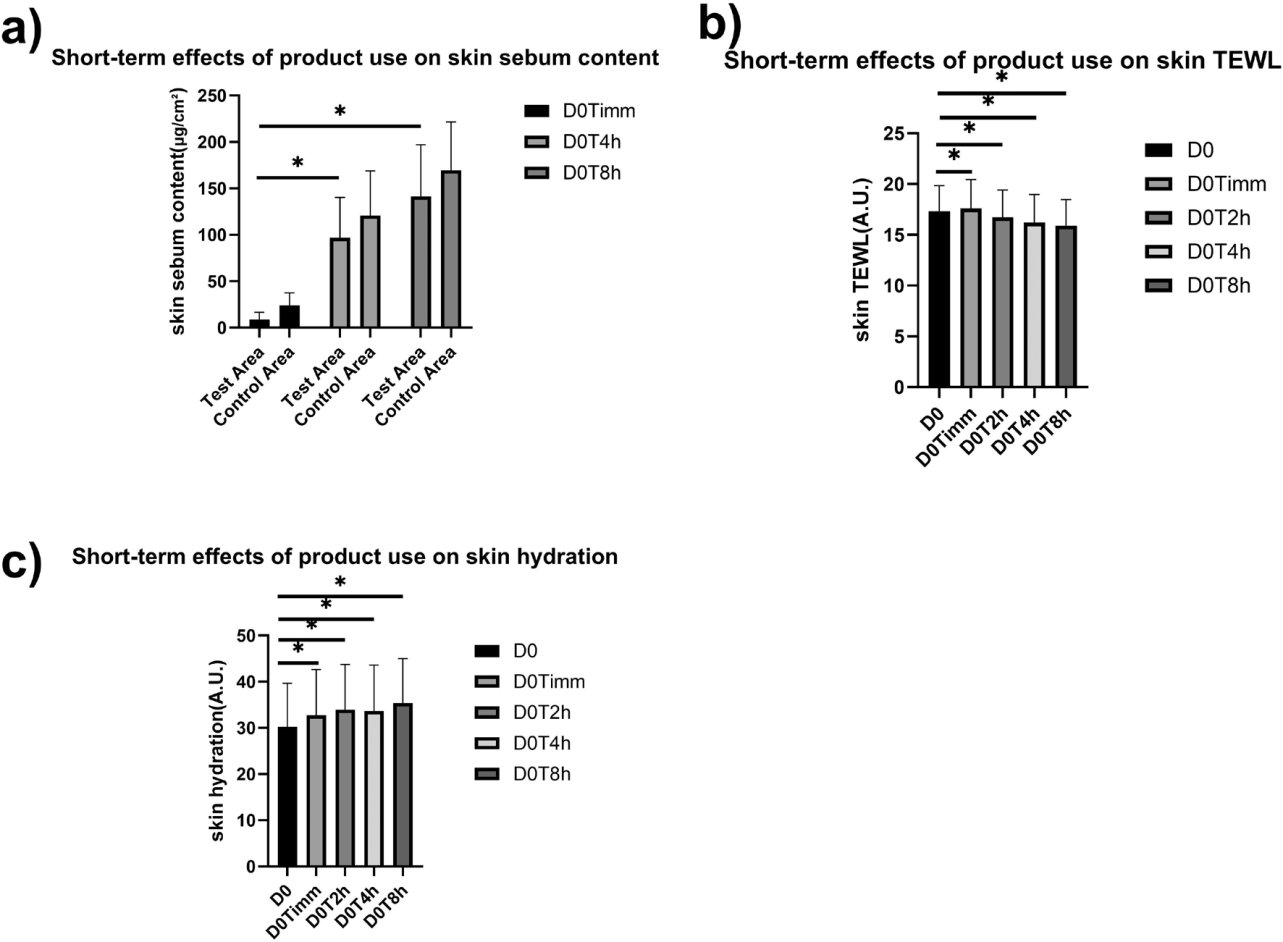
Short‐term effects of product use on skin conditions: (a) skin sebum, (b) skin TEWL, and (c) skin hydration.

Compared to before using the product, 4 h after product use, the skin sebum content in the test area significantly (*p* < 0.05) increased by 994.61%, while the skin sebum content in the control area significantly (*p* < 0.05) increased by 406.11% (Figure 1a). 8 h after product use, the skin sebum content in the test area significantly (*p* < 0.05) increased by 1499.72%, while the skin sebum content in the control area significantly (*p* < 0.05) increased by 608.93%. In addition, compared to the blank control area, the change in skin sebum levels at the product application site showed a statistically significant difference both 4 h and 8 h after application.

Compared to baseline, immediately after application, TEWL in the cheek test area increased by 1.62% (Figure 1b). Two hours after application, TEWL in the cheek test area decreased significantly (*p* < 0.05) by 3.34%. Four hours after application, TEWL in the cheek test area decreased significantly (*p* < 0.05) by 6.40%. Eight hours after application, TEWL in the cheek test area decreased significantly (*p* < 0.05) by 8.06%.

Compared to baseline, immediately after application, skin hydration in the cheek test area increased significantly (*p* < 0.05) by 8.52% (Figure 1c). Two hours after application, skin hydration in the cheek test area increased significantly (*p* < 0.05) by 12.39%. Four hours after application, skin hydration in the cheek test area increased significantly (*p* < 0.05) by 11.37% (*p* < 0.05). Eight hours after application, skin hydration in the cheek test area increased significantly (*p* < 0.05) by 17.15%.

**FIGURE 2:**
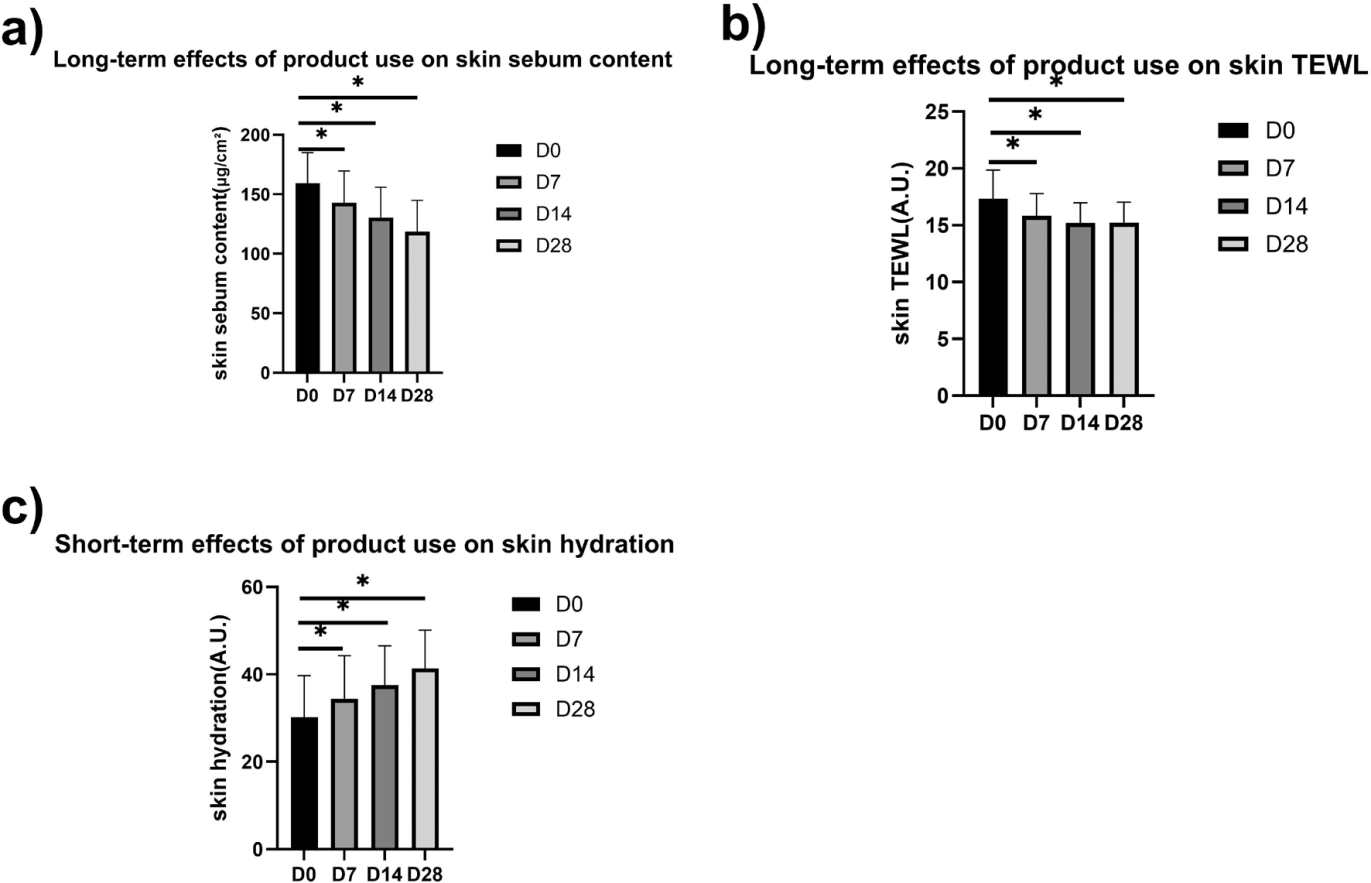
Long‐term effects of product use on skin conditions: (a) skin sebum, (b) skin TEWL, and (c) skin hydration.

Compared to baseline, 7 days after application, skin sebum in the forehead test area decreased significantly (*p* < 0.05) by 10.30% (Figure 2a). Fourteen days after application, skin sebum in the forehead test area decreased significantly (*p* < 0.05) by 18.02%. 28 days after application, skin sebum in the forehead test area decreased significantly (*p* < 0.05) by 25.64%.

Compared to baseline, 7 days after application, TEWL in the cheek test area decreased significantly (*p* < 0.05) by 8.66% (Figure 2b). Fourteen days after application, TEWL in the cheek test area decreased significantly (*p* < 0.05) by 12.32%. 28 days after application, TEWL in the cheek test area decreased significantly (*p* < 0.05) by 12.04%.

Compared with baseline, 7 days after application, the skin hydration of cheek test area significantly increased by 13.95% (Figure 2c). Fourteen days after application, the skin hydration of cheek test area significantly increased by 24.17%. 28 days after application, the skin hydration of cheek test area significantly increased by 24.17%.

### 3.2 Dermatologist Evaluation

**FIGURE 3:**
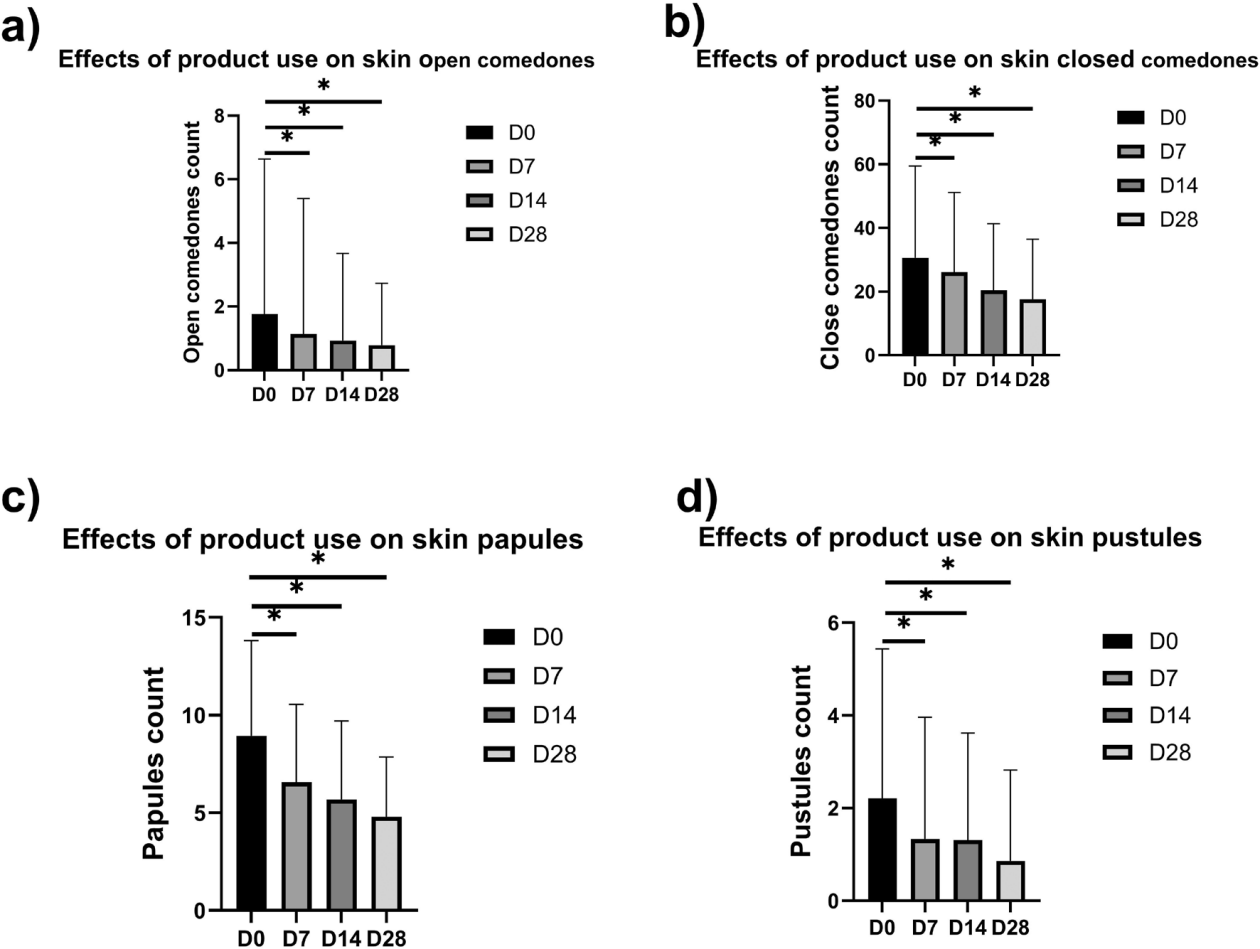
Effects of product use on skin acne‐related indicators: (a) open comedones, (b) closed comedones, (c) skin papules, and (d) skin pustules.

Compared to baseline, 28 days after application, the number of open comedones decreased significantly (*p* < 0.05) by 55.41% (Figure 3a), the number of closed comedones decreased by 42.58% (Figure 3b), the number of papules decreased by 46.28% (Figure 3c), and the number of pustules decreased by 61.29% (Figure 3d).

**FIGURE 4:**
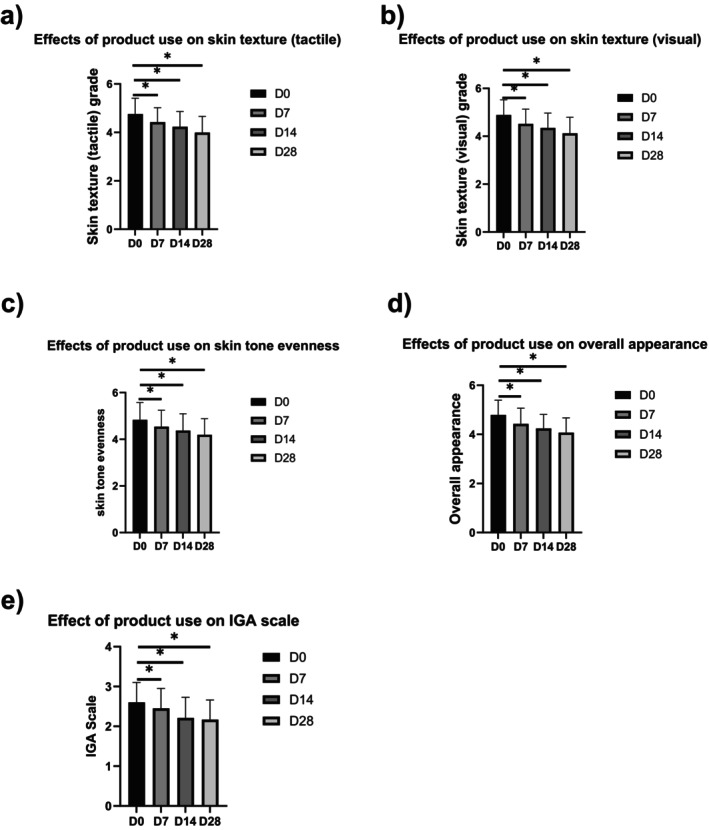
Efficacy of product use on skin appearances: (a) skin texture (tactile), (b) skin texture (visual), (c) skin tone evenness, (d) overall appearance of skin, and (e) IGA scale.

Compared with baseline, 28 days after application, the score of skin texture (tactile) significantly by 16.25% (Figure 4a), the score of skin texture (visual) significantly decreased by 15.82% (Figure 4b), the score of skin tone evenness significantly decreased by 13.30% (Figure 4c), the score of overall appearance of skin significantly decreased by 13.30% (Figure 4d), the score of IGA scale significantly decreased by 16.51% (Figure 4e).

## 4 Discussion

Acne is a complex dermatological disorder driven by multiple contributing factors. A key aspect of its pathogenesis is the overproduction of sebum coupled with hypertrophy of the sebaceous glands, which results in the accumulation of keratin within the follicular walls, ultimately leading to the development of microcomedones. The use of topical active agents, including salicylic acid, for acne management has been extensively documented in the literature [6]. However, the relationship between facial cleansing practices and acne remains incompletely understood. Emerging evidence suggests that active compounds, such as salicylic acid, incorporated into cleanser formulations can effectively permeate the skin and deliver therapeutic benefits [7]. Our study demonstrates that a cleanser containing salicylic acid, glucuronolactone, hectorite, ceramides, and niacinamide is effective in reducing acne lesions and modulating sebum secretion. Salicylic acid exerts its action by dissolving intercellular lipids, thereby disrupting comedones, and additionally suppresses the growth of *Propionibacterium acnes* , the bacterium implicated in acne pathogenesis [8]. Furthermore, salicylic acid inhibits sebum secretion by suppressing the AMPK/SREBP‐1 pathway in sebocytes [9]. Gluconolactone, on the other hand, functions by diminishing the adhesion between corneocytes in the basal layer, facilitating desquamation and preventing follicular blockage. Notably, gluconolactone offers a gentler alternative with fewer adverse effects compared to benzoyl peroxide [10]. In addition, hectorite can absorb excess sebum [11]. Furthermore, niacinamide contributes to sebum regulation and possesses anti‐inflammatory properties, enhancing its therapeutic potential in acne treatment [12].

Transepidermal water loss (TEWL) is an important indicator of the skin barrier's integrity [13]. Studies have consistently shown that higher TEWL values are strongly associated with a compromised skin barrier, whereas lower TEWL values are indicative of healthier skin [14]. Previous studies have shown that cleansers can lead to an increase in TEWL, resulting in damage to the skin barrier [15, 16]. In contrast, our study's data indicated a significant improvement in the facial TEWL index. This may be due to the inclusion of ceramide EOP, ceramide NP, and ceramide AP in the formula, which have structures identical to those found naturally in the skin barrier. A previous study has reported that ceramide EOP, ceramide NP, and ceramide AP play crucial roles in maintaining skin lipid structure, thereby effectively enhancing the skin barrier function [17]. On the other hand, the product also contains niacinamide, which a previous study has shown not only stimulates the production of various skin physiological lipids, such as ceramides, fatty acids, and cholesterol [18], but also increases the protein content of the barrier layer [19].

Skin tone affects the overall appearance of the skin. It is not only related to skin roughness but is also closely connected to the melanin content in the skin. The results of this study indicate that the cleanser not only significantly improves skin tone but also greatly enhances skin texture. This may be due to the inclusion of salicylic acid, which has exfoliating properties [20] that help reduce skin roughness and improve skin texture. On the other hand, niacinamide has been proven to inhibit melanin transfer, thereby improving skin tone [21].

This study also has some limitations. Currently, only skin‐related physiological parameters were measured, without further investigation into the molecular‐level changes in the skin after product use. The next step could involve exploring the overall changes using lipidomics and proteomics.

## 5 Conclusion

In summary, an amino acid cleanser containing salicylic acid, glucuronolactone, and ceramides has anti‐acne, repairing, soothing, and texture‐improving properties, offering multidimensional improvement for acne and oily‐prone skin conditions.

## Author Contributions

Yunfei Ai Levin: writing – review and editing. Yansi Lyu: conceptualization; writing – preparation of first draft. Xiaofeng He: methodology.

## Ethics Statement

This study was conducted in accordance with the Declaration of Helsinki and approved by the SGS Ethics Committee (protocol code: SHCPCH24005000‐01). All subjects were informed and gave their consent before enrollment.

## Conflicts of Interest

The authors declare no conflicts of interest.

## Data Availability

The data that support the findings of this study are available from the corresponding author upon reasonable request.

## References

[1] J. U. R. Blaak , S. Grabmann , I. Simon , J. Blaak , T. Callaghan , and P. Staib , “Five Dimensions of Cleansing: A Holistic View on the Facets and Importance of Skin Cleansing,” International Journal of Cosmetic Science 45, no. 5 (2023): 557–571. 37367943 10.1111/ics.12879

[2] A. Yokoi , K. Endo , T. Ozawa , et al., “A Cleanser Based on Sodium Laureth Carboxylate and Alkyl Carboxylates Washes Facial Sebum Well but Does Not Induce Dry Skin,” Journal of Cosmetic Dermatology 13, no. 4 (2014): 245–252. 25399616 10.1111/jocd.12118

[3] G. Peterson , S. Rapaka , N. Koski , M. Kearney , K. Ortblad , and L. Tadlock , “A Robust Sebum, Oil, and Particulate Pollution Model for Assessing Cleansing Efficacy of Human Skin,” International Journal of Cosmetic Science 39, no. 3 (2017): 351–354. 27797421 10.1111/ics.12378

[4] A. Seweryn , “Interactions Between Surfactants and the Skin–Theory and Practice,” Advances in Colloid and Interface Science 256 (2018): 242–255. 29685575 10.1016/j.cis.2018.04.002

[5] T. Stringer , A. Nagler , S. J. Orlow , and V. S. Oza , “Clinical Evidence for Washing and Cleansers in Acne Vulgaris: A Systematic Review,” Journal of Dermatological Treatment 29, no. 7 (2018): 688–693. 29460655 10.1080/09546634.2018.1442552

[6] A. Akhavan and S. Bershad , “Topical Acne Drugs: Review of Clinical Properties, Systemic Exposure, and Safety,” American Journal of Clinical Dermatology 4 (2003): 473–492. 12814337 10.2165/00128071-200304070-00004

[7] M. A. Davies , “Salicylic Acid Deposition From Wash‐Off Products: Comparison of in Vivo and Porcine Deposition Models,” International Journal of Cosmetic Science 37, no. 5 (2015): 526–531. 25899428 10.1111/ics.12229

[8] M. A. Blaskovich , A. G. Elliott , A. M. Kavanagh , M. A. T. Blaskovich , S. Ramu , and M. A. Cooper , “In Vitro Antimicrobial Activity of Acne Drugs Against Skin‐Associated Bacteria,” Scientific Reports 9, no. 1 (2019): 14658. 31601845 10.1038/s41598-019-50746-4 PMC6787063

[9] J. Lu , T. Cong , X. Wen , et al., “Salicylic Acid Treats Acne Vulgaris by Suppressing AMPK/SREBP 1 Pathway in Sebocytes,” Experimental Dermatology 28, no. 7 (2019): 786–794. 30972839 10.1111/exd.13934

[10] M. J. Hunt and R. S. Barnetson , “A Comparative Study of Gluconolactone Versus Benzoyl Peroxide in the Treatment of Acne,” Australasian Journal of Dermatology 33, no. 3 (1992): 131–134. 1303072 10.1111/j.1440-0960.1992.tb00100.x

[11] F. D. Sarruf , V. J. P. Contreras , R. M. Martinez , M. V. R. Velasco , and A. R. Baby , “The Scenario of Clays and Clay Minerals Use in Cosmetics/Dermocosmetics,” Cosmetics 11, no. 1 (2024): 7.

[12] Z. D. Draelos , A. Matsubara , and K. Smiles , “The Effect of 2\% Niacinamide on Facial Sebum Production,” Journal of Cosmetic and Laser Therapy 8, no. 2 (2006): 96–101. 16766489 10.1080/14764170600717704

[13] M. Green , N. Kashetsky , A. Feschuk , and H. I. Maibach , “Transepidermal Water Loss (TEWL): Environment and Pollution—A Systematic Review,” Skin Health and Disease 2, no. 2 (2022): e104. 35677917 10.1002/ski2.104 PMC9168018

[14] M. Green , A. M. Feschuk , N. Kashetsky , and H. I. Maibach , ““Normal” TEWL‐How Can It Be Defined? A Systematic Review,” Experimental Dermatology 31, no. 10 (2022): 1618–1631. 35753062 10.1111/exd.14635

[15] J. Spoo , W. Wigger‐Alberti , U. Berndt , T. Fischer , and P. Elsner , “Skin Cleansers: Three Test Protocols for the Assessment of Irritancy Ranking,” Acta Dermato‐Venereologica 82, no. 1 (2002): 13. 12013190 10.1080/000155502753600812

[16] J. Eo , Y. K. Seo , J. H. Baek , et al., “Facial Skin Physiology Recovery Kinetics During 180 Min Post‐Washing With a Cleanser,” Skin Research and Technology 22, no. 2 (2016): 148–151. 26100540 10.1111/srt.12241

[17] A. Schroeter , A. Eichner , J. Mueller , et al., “The Importance of Stratum Corneum Lipid Organization for Proper Barrier Function,” in Percutaneous Penetration Enhancers Chemical Methods in Penetration Enhancement: Drug Manipulation Strategies and Vehicle Effects (Springer, 2015), 19–38.

[18] O. Tanno , Y. Ota , N. Kitamura , et al., “Effects of Niacinamide on Ceramide Biosynthesis and Differentiation of Cultured Human Keratinocytes,” Journal of Investigative Dermatology 4, no. 108 (1997): 643.

[19] W. Gehring , “Nicotinic Acid/Niacinamide and the Skin,” Journal of Cosmetic Dermatology 3, no. 2 (2004): 88–93. 17147561 10.1111/j.1473-2130.2004.00115.x

[20] S. N. Wi , J. Niewska , S. Klasik‐Ciszewska , and L. K. Duda‐Grychto , “Salicylic Acid and Its Use in Cosmetology,” Aesthetic Cosmetology and Medicine 12, no. 3 (2023): 91–95.

[21] Y. C. Boo , “Mechanistic Basis and Clinical Evidence for the Applications of Nicotinamide (Niacinamide) to Control Skin Aging and Pigmentation,” Antioxidants 10, no. 8 (2021): 1315. 34439563 10.3390/antiox10081315 PMC8389214

